# Attention-based bidirectional-long short-term memory for abnormal human activity detection

**DOI:** 10.1038/s41598-023-41231-0

**Published:** 2023-09-02

**Authors:** Manoj Kumar, Anoop Kumar Patel, Mantosh Biswas, S. Shitharth

**Affiliations:** 1 JSS Academy of Technical Education, Noida, India; 2 National Institute of Technology Kurukshetra, Kurukshetra, India; 3 Kebri Dehar University, KebriDehar, Ethiopia

**Keywords:** Data processing, Computer science

## Abstract

Abnormal human behavior must be monitored and controlled in today’s technology-driven era, since it may cause damage to society in the form of assault or web-based violence, such as direct harm to a person or the propagation of hate crimes through the internet. Several authors have attempted to address this issue, but no one has yet come up with a solution that is both practical and workable. Recently, deep learning models have become popular as a means of handling massive amounts of data but their potential to categorize the aberrant human activity remains unexplored. Using a convolutional neural network (CNN), a bidirectional long short-term memory (Bi-LSTM), and an attention mechanism to pay attention to the unique spatiotemporal characteristics of raw video streams, a deep-learning approach has been implemented in the proposed framework to detect anomalous human activity. After analyzing the video, our suggested architecture can reliably assign an abnormal human behavior to its designated category. Analytic findings comparing the suggested architecture to state-of-the-art algorithms reveal an accuracy of 98.9%, 96.04%, and 61.04% using the UCF11, UCF50, and subUCF crime datasets, respectively.

## Introduction

One of the major challenges confronting the area of computer vision due to the rising number of cameras and surveillance systems in megacities is the development of autonomous approaches for analyzing video-based scenarios. Activities that may indicate possible security hazards to persons, places, or items are of particular relevance. The majority of the methods used to study the subject have been centered on recognizing the unique actions people engage in unusual activities. These unusual activities may be in the category of crime and that is of our interest ^1^. Situations frequently become complicated as a result of numerous relationships between humans or between individuals and environmental objects. In these situations, context information has recently been included to enhance the autonomous systems' capacity for interpretation ^2^. Although there are many different types of suspicious activity, the current research focuses on those that cause harm to people or objects in solitary situations or low-light situations. When criminal conduct occurs in such circumstances, it is common to see suspects stalking their victims before the ultimate act of assault. Such a set of events could indicate strange activities, which may be controlled using an early warning system.

Even having a remarkable advancement in camera movements, complex backgrounds, occlusions, and varying levels of illumination, the task of human action recognition is a challenge. Action detection and recognition have a lot of applications in the areas such as industrial monitoring, cloud environment, violence detection, virtual reality, and person identification ^3, 4^. When identifying various human actions in video streams, spatial and temporal information is essential. For describing the appropriate action in the video, the majority of methods utilized handmade features that are used to transform a signal into 3-D aspects of dynamic motion. Because of the movement style and the extensive backdrop clutter, the handcrafted-backed structures technique in action identification is mostly database-based and fails to fulfil the universal scenario. In order to capture reliable information, handcrafted features, and representative motion features are progressively improved from 2 to 3D spatiotemporal features ^5^.

For learning high-level distinguishing characteristics and creating complete systems for action and behavior detection based on the video, deep learning (DL) is currently the most popular and commonly utilized technique ^6^. Simple CNNs are used in convolution operations in the current DL methods for human action recognition (HAR), which use pre-trained models to train the characteristics from video-stream. These convolutional layers learn and extract spatial characteristics to be used in the training of a classification model. Common CNN models underperform hand-crafted features when working with sequential data^7^. AlexNet, ResNet, and VGG are just a few examples of conventional CNN models that can study spatial properties from a solitary input copy. These mock-ups work well for taking geographical data, but they struggle to capture temporal data, which is crucial for Abnormal Human Activity Recognition (AbHAR) to catch motion data in a video series. Dai et al. ^8^ have proposed a learning algorithm using coupled characteristics such as spatial and temporal features which are extracted using CNN and the LSTM respectively. The two-stream method is necessary to develop separate modules for the video-based high-level AbHAR algorithms because they study spatial and sequential aspects in filmed arrangements by combining processes to collect dynamic data in sequential data^9^. RNNs have recently been used to address spatiotemporal difficulties, with the LSTM especially created for video sequences to study and interpret the chronological aspects of HAR in video investigation systems^10^. To address the current difficulties and limitations of the HAR, the majority of researchers have devised a two-stream technique for activity recognition that combines chronological and spatial data and fuses them to train the model.

Consequently, it is still difficult to precisely recognize action in real-life recordings due to a lack of data on motion, style, and backdrop clutter, all of which are necessary for the correct identification of human movements. Conventional approaches failed to address these issues due to difficulties in managing continuous activities, and difficulties in modeling congested situations due to complex contexts ^11^. LSTMs and GRU (Gated Recurrent Units) were able to explicitly address AbHAR’s sequence learning issues by taking sequence-specific information into account, which is required to ensure seamless transitions between frames. As a means of solving this problem, we present an innovative attention-based AbHAR system that can study spatiotemporal properties and especially focus on distinguishing prompts in the long-term stream to recognize activity in video frames, making it well-suited for use in a surveillance system. This system employs a Deep Convolutional Neural Network (DCNN) with attention blocks to enhance the learned features, while a Bi-LSTM with attention weights allows it to narrow in on the most pertinent information from the input frame sequence for motion detection. The suggested method uses a combination of CNN's convolution operation, which extracts spatial information, and the Bi-LSTM, which processes this information to produce content that better recommends actions for humans.

Extracting the high-level distinguishing information from a frame stream and passing it on to update the attention weights for specific signals in sequence, the attention mechanism must process every fifth frame of the video. As can be shown from the experiments, the suggested technique is better suited for the AbHAR for the investigation video streams because of these characteristics. The use of a CNN with remaining attention blocks to improve the topographies is the main contribution of the proposed AbHAR system. A more accurate depiction of human activity in surveillance footage for investigative purposes is achieved by combining conventional approaches with the skipping connection concept. To better understand the temporal and spatial relationships present in sequential data, we suggest combining a deep Bi-LSTM with an attention mechanism. To recognize human activities in a series, the attention weight is adjusted using the learned global features. By experimentally evaluating the proposed AbHAR system UCF11, UCF50, and UCF crime action datasets, we conclude that it has a good performance of activity recognition of 98.9%, 96.04%, and 61.04%, respectively. The proposed system achieves superior performance to state of art approaches, which bodes well for its viability and use in real-world surveillance settings.

The main contribution of the paper is:

- *Addressing the Need for Real-Time Monitoring* In order to prevent injury to society, both physical and web-based forms of violence, the study acknowledges the significance of monitoring and regulating aberrant human activities in the modern era. It fills a void in the existing literature by highlighting the need for a solution that can function in real-time scenarios.
- *Utilizing Deep Learning Models* The research emphasizes the use of deep learning models, which are renowned for their efficient handling of large datasets. It highlights the existing research gap regarding the application of deep learning models to the classification of abnormal human activity.
- *Proposed Framework* This article describes a novel framework that integrates various deep learning techniques to detect aberrant human behavior. It combines a convolutional neural network (CNN) for spatial feature extraction, a bidirectional long short-term memory (Bi-LSTM) for capturing temporal dependencies, and an attention mechanism to focus on particular spatiotemporal characteristics in unprocessed video streams.
- *Accurate Classification of Aberrant Human Activity* Processing real-time video demonstrates the efficacy of the proposed framework in accurately classifying aberrant human activity. When evaluated using the UCF11, UCF50, and subUCF crime datasets, respectively, the architecture obtains high accuracy rates of 98.9%, 96.04%, and 61.04%. These results are compared to state-of-the-art algorithms, which further demonstrate the effectiveness of the proposed method.

The remaining sections of this paper are structured as follows. The second section “Literature survey” examines prior relevant studies. Section “Proposed methodology” delves into the context model and the inference stage in further depth is the proposed methodology. Section “Result and discussion” is the result and discussion which presents the validation mechanism as well as the experimental outcomes. Finally, section “Conclusion” wraps off and sketches out future works.

## Literature survey

Deep learning-based methods entirely replace handcrafted features-based methods in Abnormal Human Activity Recognition (AbHAR). In AbHAR, CNN-based approaches are the most frequent. A CNN ^12^ is a customized neural network that uses the image’s structural information to build the neural network. CNN, like a traditional neural network, is made up of learnable weights. The dot product of input data with some random weights for each neuron is the first step in the training process. Backpropagation is used to update these weights. The network is made up of a single differentiable score function that generates a classification class as a result of the gradient from the raw picture pixels. They also contain a loss function that is used to reduce the score to the lowest possible level.

CNNs are a kind of deep learning model that apply a grading of progressively complex structures to raw input images using trainable filters and neighborhood merging procedures. CNNs have been found to outperform humans on visual object recognition tasks when trained with adequate regularisation ^13^. Furthermore, CNNs are unaffected by certain variables such as stance, lighting, and clutter ^12^.

In various applications, such as video ^14, 15^ image, speech, and signal processing, it is utilized to learn a structure of features and their relative importance ^16^. As a result, feedforward neural network models can attain state of art object classification accuracy, occasionally outperforming human presentation. These models are accomplished using large datasets of labeled data and multilayer neural network constructions. Numerous 3D Deep Learning and 3D CNNs for HAR were introduced by Baccouche et al. ^17^ and Latah ^18^. On the KTH dataset, his strategies perform well. In addition, Deldjoo et al. ^19^ just published a paper with successful results on deep learning for movie selection. However, various flaws have been discovered. To capture all of the relationships between input samples, sharing parameters throughout time is insufficient. Furthermore, local connectivity restricts the output to the small scale of surrounding input samples. RNNs have had a lot of success with sequence labeling and prediction tasks like language modeling and handwriting recognition so far. As a result, numerous types of concealed units for RNN have been employed to address a range of issues with excellent results in several applications using sequential or temporal data ^19^. In diverse tasks such as video captioning ^20, 21^, speech recognition ^22^, and handwriting recognition, the LSTM units presented by Hochreiter and Schmidhuber ^23^ are used with RNN. As a result, several LSTM network topologies are developed to maximize a variety of applications. Alex Graves ^22^ demonstrated bidirectional LSTM (Bi-LSTM) networks to classify phoneme framewise, and It’s also put to use in the construction of a multi-stream framework for real-time speech detection in ongoing conversations. Hasim Sak suggested LSTM Projected for Large Scale Acoustic Modeling ^24^. Empirical evidence demonstrates LSTM's efficacy in simulating long-term temporal dependency across a range of computer vision tasks. A novel system proposed by Ansari et al. ^25^ to identify and mitigate the shoplifting activity in megastore using inception and LSTM framework on self-created dataset which perform 91.8% accuracy. An another approach suggested by Ansari et al.^26^ to detect shoplifting using optical flow and gradient information, which detect salient motion feature and accurately identify the activity. Dwivedi et al. ^27^ was proposed an novel approach to detect suspicious activity using pretrain network and LSTM on a dataset where activities are collected from eleven benchmark dataset.

The model may emphasize key information by assigning varying weights to various parts of the visual content, to the attention mechanism. Since then, the attention mechanism has been extensively implemented in visual comprehension, with impressive results in a variety of tasks including object acknowledgment, image captioning, image query responding, and saliency detection. The earliest application of attention to the NLP problem was by Bahdanau et al. ^28^, who utilized it to conduct concurrent conversion and alignment in machine translation jobs. To forecast the spread of influenza, Zhu et al. ^29^ developed neural networks using simple attention blocks. Additionally, many attention-based RNN model variants have arisen in the NLP community. The widespread adoption of the attention mechanism in fields as diverse as multimedia recommendation^30^ and medical diagnosis^31^ demonstrates its versatility and utility in a wide range of machine-learning contexts. Human pose estimation using stacked hourglass networks for feature extraction was planned by Chun et al.^32^. Author^33^ presents a deep learning model to identify anomalous events, and it is tested using raw footage from the UCF crime dataset. The pre-train network DenseNet was used to excerpt the features. Using spatial characteristics and temporal information retrieved by Bi-LSTM, this model was able to categorize atypical activity and identify the type of aberrant behavior. This setup combines the temporal properties of the bidirectional LSTM network with the spatial abilities of the CNN. When applied to action recognition, the suggested model with bi-LSTM showed substantial improvement.

Precisely, the mentioned work concentrates on developing a deep neural network and recurrent neural network using deep features and handcrafted features to have an efficient classification tool for cooperative/noncooperative activity. The proposed work is based on an attentive recurrent architecture with pre-train CNN that provides excellent discrimination between various human behaviors such as normal or abnormal in video surveillance.

## Proposed methodology

**Figure 1:**
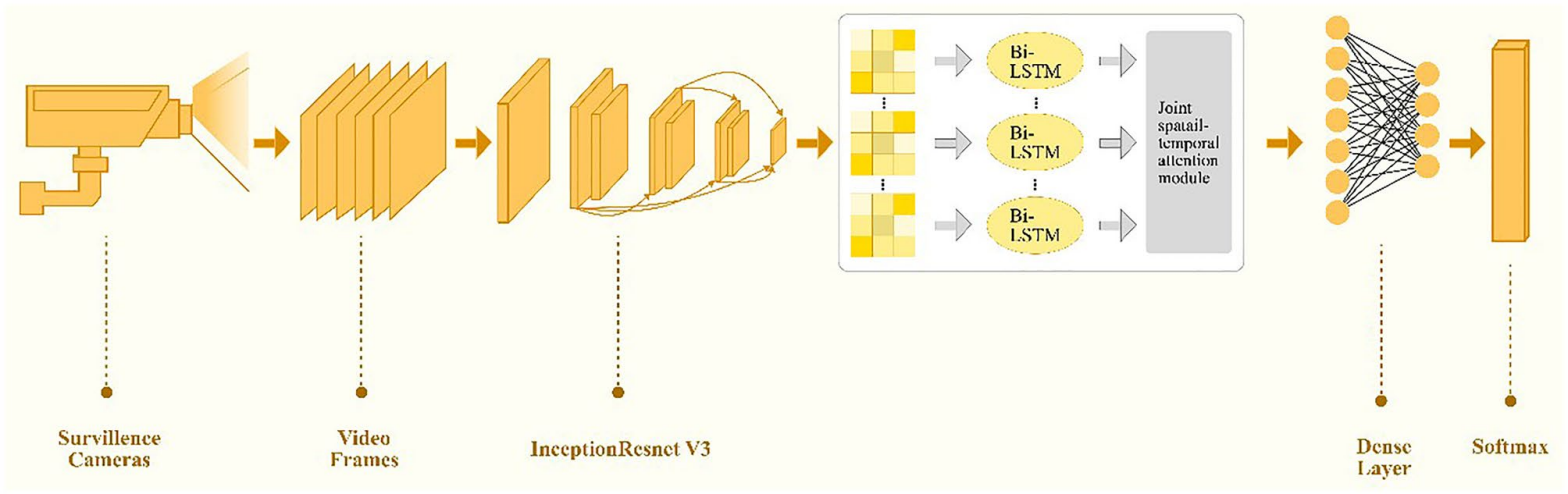
Proposed model.

As given in Fig. 1, The proposed method initiates with spatial feature extraction and moves on to temporal feature analysis also shown in Fig. 1. For static feature extraction, each frame uses the recent pre-trained model InceptionResnet-V3. In InceptionResnet-V3, the final pooling layer makes use of transfer learning to automatically extract features from video frames for visual data. Pre-train network transfers the output to the first Bi-LSTM layer as its input.

The input for the attention module is produced by the suggested spatiotemporal feature extraction module. By stacking numerous Residual Attention Bi-LSTM blocks, the feature set is created. Each block transfers the output of the preceding block to the first Bi-LSTM of its input. Let $c_{t}^{\left( i \right)}$ be the ith the temporal feature vector produced by the Bi-LSTM at time t. The attention layer generates a background vector $h_{t}$ for $c_{t}^{\left( i \right)}$ at time t by allocating the attention weights $a_{t}^{\left( i \right)}$.with the help of Eq. (1), the context vector can be considered.

1 $$h_{t} = \mathop \sum \limits_{i = 1}^{M} a_{t}^{\left( i \right)} c_{t}^{\left( i \right)}$$

The total number of features is denoted by M. An activation function is applied to the first Bi-LSTM layer’s hidden state $h_{t}$ to produce the relevant score $s_{t}^{\left( i \right)}$,which is described in Eq. (2).

2 $$s_{t}^{\left( i \right)} = {\text{tanh}}\left( {Wh_{t} + b} \right)$$

The time t for a feature I is denoted by $s_{t}^{\left( i \right)}$. The model learned the weight and bias parameters denoted by W and b respectively. Activation function $\tanh ()$ represents the hyperbolic tangent. Finally, the attention module formulates the attention weight $a_{t}^{\left( i \right)}$ as an Eq. (3).

3 $$a_{t}^{\left( i \right)} = \frac{{{\text{exp}}\left( {w_{t}^{\left( i \right)} s_{t}^{\left( i \right)} } \right)}}{{\mathop \sum \nolimits_{j = 1}^{M} exp\left( {w_{t}^{\left( i \right)} s_{t}^{\left( i \right)} } \right)}}$$

where $w_{t}^{\left( i \right)}$ is the model weight learned at time t for feature i. In time step t, the denominator is the aggregate of all features’ weighted scores multiplied by their respective relevant scores. The remaining unit is shaped by generating cut-offs amid each Bi-LSTM and consideration layer. Combining the acquired non-linear plotting F(x) with the uniqueness mapping x helps the network to minimize information loss as Eq. (4).

4 $${\text{Y}} = {\text{F}}\left( {\text{x}} \right) + {\text{x}}$$

where y and x are the output and input of the remaining block.

### Attention mechanism

Even though Bi-LSTM networks excel at collecting long-range dependencies, they are unable to identify the specific input series elements that are essential for delivering a more accurate classification. This problem can be resolved by using the attention technique. The output of Bi-LSTM network’s $h_{1} , \;h_{2} ,\;h_{3}$ and $h_{n}$ vectors are fed to the attention layer, where they are subsequently encoded into the information vectors $x_{1} , \;x_{2} ,\;$ $x_{3}$ and $x_{n}$ by the attention encoders. The context vectors are computed in this procedure using the weighted sum of the encoder RNN output. Equation (5) is used to calculate the context vectors $c_{1} ,\;c_{2} , \;c_{3}$ and $c_{n}$.

5 $$c_{t} = \mathop \sum \limits_{t = 0}^{n} a_{t} \cdot x_{t}$$

The encoded information vector ($c_{t}$) and the attention score ($a_{t}$) are both used. The attention scores are calculated using (6) and (7). In (7), the prior cell state vector is represented by d, the feedforward network is defined by the function $F_{att}$, and the encoded information vector is designated by $x_{t}$ (t−1).

6 $$softmax\left( {a_{t} } \right) = \frac{{exp\left( {o_{t} } \right)}}{{\mathop \sum \nolimits_{t = 0}^{n} exp\left( {o_{t} } \right)}}$$

7 $$o_{t} = F_{att} \left( {x_{t} \cdot d_{t - 1} } \right)$$

The context vectors $c_{t}$ the output of the prior time step, $y_{t - 1}$ and the prior cell state vector, $d_{t - 1}$ are all used to determine the output of this attention layer at every time t.

## Result and discussion

Here, we describe the datasets and experimental setup we employed before comparing our proposed network to several existing approaches that have been deemed state-of-the-art in education to demonstrate the superiority of the proposed model. The results of the experimental ablation on various network components are then described.

### Dataset

We performed tests on the UCF-11, UCF-50, and SUB-UCF crime datasets, three of the most difficult multi-person human activity datasets.

UCF11: There are 11 different action genres in it, including biking, basketball shooting, diving, golf swinging, juggling soccer, horseback riding, trampoline jumping, volleyball spiking, tennis swinging, and walking a dog. Due to the wide variations in camera motion, item look and posture, viewpoint, object scale, radiance conditions, messy context, etc., this data set is quite difficult to work with. There are a total of 25 categories for the videos, and at least four action clips may be found in each. In general, the videos that make up a single set will share some commonalities, such as the same actor, a similar environment, a similar point of view, etc.

UCF50: UCF50 is a data set for action recognition built from 50 different types of realistic action videos pulled from YouTube. Most action recognition data sets are unrealistic since they were created with actors performing in a studio. This dataset for action recognition is realistic and extremely challenging because of the wide variety in camera movements, item looks and postures, object scale, viewpoint, crowded background, lighting conditions, etc. Each of the 50 categories has its own set of 25 subcategories, and each subcategory has more than 4 action videos. Similarities between the videos in a set can include the presence of a common character, setting, or point of view.

Sub-UCF-crime: To evaluate our approach, we generate a new big dataset we call UCF-Crime. This compilation features hours of raw surveillance footage from five different strange situations, including fire, abuse, arrest, assault, and fighting. These peculiarities were selected because of their potentially devastating effects on public security.

### Implementation details and hyper-parameter settings

To showcase our proposed architecture, we experimented with the most commonly downloaded datasets i.e. UCF 11, UCF 50, and Sub-UCFcrime. Python 3.7, Anaconda/3, and CUDA/10 are installed on a Windows server with an i5 CPU, 2 GB GPU, and 8 GB RAM. In addition to the aforementioned parameters, the Python libraries Tensorflow-Keras, OpenCV, matplotlib, os, math, and NumPy are employed. As shown in Table 1, we have trained the system for 80 epochs using hyperparameters.

**Table 1:** Hyperparameter setting used in the experiment.

Hyperparameter	Value
Optimizer	Adam
Loss Function	Categorical_cross-entropy
Batch size	16
Number of Epochs	80
Learning rate	0.001
Decay	1e−6
Image resize	50 × 50
Stride	(1,1)

### Performance metrics

Accuracy, precision, recall, confusion matrix and class-wise correctness are used to assess the presence of the planned system with respect to the binary classification problem at hand. True Positive (TP), True Negative (TN), False Positive (FP), and False Negative (FN) must be defined before defining these concepts. Assume that the two classes in a problem of binary classification are positive and negative. TP refers to the classification of a sample as positive. FP refers to a sample that has been incorrectly categorized as positive when it belongs to the negative class. In a similar manner, TN refers to a sample that has been correctly categorized as a member of the negative class. FN refers to a sample that is classed as negative despite belonging to the positive class.

Accuracy: It is the quantity of properly classified samples to the total amount of samples.

8 $$Accuracy = \frac{TP + TN}{{TP + TN + FP + FN}}$$

Precision: The amount of properly recognized Positive samples to the total quantity of Positive examples determines precision (either correctly or incorrectly). Exactness is the degree to which a model correctly identifies a sample as positive.

9 $$Precision = \frac{TP}{{TP + FP}}$$

Recall: The Recall is intended as the amount of properly recognized Positive examples compared to the entire number of Positive examples. Recall measures the model’s capacity to recognize Positive samples. As memory grows, an increasing number of positive samples are detected.

10 $$Recall\left( {sensitivity} \right) = \frac{TP}{{TP + FN}}$$

### Model’s training

For model training, 80 iterations of the Adam optimizer were utilized. Smaller batch sizes are chosen since they improve the model’s test accuracy and expedite the network's capacity to learn. Adam’s optimization has a 0.001 percent learning rate. Adam is utilized to train the model since it informs the network weight repeatedly based on the training dataset. The results of adaptive moment estimation in Adam. The dataset’s validation loss is the condition for epoch termination. The training exactness is higher than the authentication correctness, because the validation data points are newly inserted unseen data points and it gives a general idea of how the proposed model will predict unseen samples.

### Result of UCF11

This is tough to work with abHAR due to camera movements, item look and posture, object gauge, lookout, crowded contextual, brightness settings, etc. The tapes are grouped into 25 groups, each containing four action clips. Similar video clips have the same performer, setting, point of view, etc. Table 2 shows that the proposed approach outperformed real event replicas^34^, motion routes ^35^, better course ^36^, and ranked clustering multi-task^37^ for this dataset, with the accuracy of 89.43%, 89.70%, 89.50%, and 98.90% respectively. Table 3 shows the class-wise accuracy of all activity which is taken for experiments. This table shows that individual class gained an accuracy not less than 96% which shows the stability of model for activities like diving, golf swinging and biking etc.

**Table 2:** Comparison with state of art approaches.

Method	Accuracy (%)
Patel et al.^34^	89.43
Meng et al. ^35^	89.70
Gharaee et al. ^36^	89.50
Dai et al. ^37^	96.90
Our proposed	98.90

**Table 3:** Class-wise accuracy of activity on dataset UCF11.

Activity	Diving	Trampoline_jumping	Soccer_juggling	Swing	Biking	Golf_swing
Accuracy	99	99	99	98	96	100
Activity	Volleyball_spiking	Tennis_swing	Walking	Horse_riding	Basketball	
Accuracy	98	96	97	98	98	

**Figure 2:**
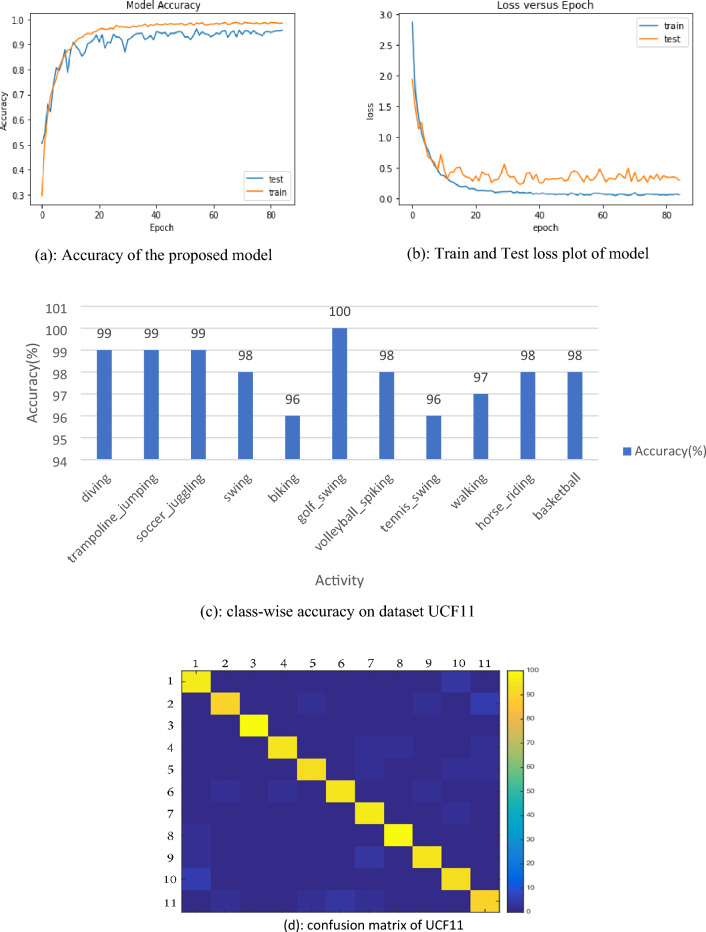
(**a**) Accuracy of the proposed model. (**b**) Train and Test loss plot of model. (**c**) Class-wise accuracy on dataset UCF11. (**d**) Confusion matrix of UCF11.

The accuracy of the model is shown in Fig. 2a and training loss vs testing loss is shown in Fig. 2b. Figure 2c and d represent the class-wise accuracy and confusion matrix respectively of all activity in dataset UCF11, where all activity recognition accuracy is more than 98.90%. Confusion matrix shows that our model accurately classifies the different classes of activity.

### Result of UCF50

UCF50 is a dataset that contains a wide spectrum of human activities, making it the most important dataset in the human activity recognition of social action appreciation in the literature. There are 50 separate activity classes and several categories have comparable characteristics in different groups. The identical activity, for example, is carried out from a different perspective.

The UCF50 dataset is exploited to compare the presented method to five activity detection algorithms: effective event models (EEM) ^38^, motion trajectories (MT) ^14^, enhanced trajectory (ET) ^39^, hierarchical clustering multi-task (HCMT)^40^, and cfeatures with ml-LSTM (CF-ML-LSTM)^41^.

Table 4 shows that the proposed approach outperformed EEM ^38^, MT ^14^, ET ^39^, HCMT ^40^ and CF-ML-LSTM ^41^ for this dataset, with an accuracy of 86.01%, 89.4%, 91.2%, 93.2%, and 94.9% respectively. From the accuracy recently reached by ML-LSTM to Effective event models, the proposed approach enhanced accuracy by 0.14–10.03% respectively. Table 5 is the class-wise accuracy of all activity used for experiment and accuracy for jump rope activity is 88% while 98% is the highest accuracy of Biking, Baseball Pitch and Punch etc.

**Table 4:** Accuracy of the previous model compared with the proposed model.

Method	Accuracy (%)
Effective event models ^38^	86.01
Motion trajectories^14^	89.40
Improved trajectory ^39^	91.20
Hierarchical clustering multi-task ^40^	93.20
Optical flow Cfeatures + ML-LSTM ^41^	94.90
Our proposed	96.04

**Table 5:** Class-wise accuracy of activity on UCF50.

Activity	Baseball Pitch	Basketball shooting	Bench Press	Biking	Billiards Shot
Accuracy	**98**	86	88	98	95
Activity	Breaststroke	Clean and Jerk	Diving	Drumming	Fencing
Accuracy	**86**	**88**	**95**	**86**	**88**
Activity	Golf Swing	Playing Guitar	High Jump	Horse Race	Horse Riding
Accuracy	**95**	**95**	**86**	**88**	**95**
Activity	Hula Hoop	Javelin Throw	Juggling Balls	Jump Rope	Jumping Jack
Accuracy	**86**	**88**	**95**	**86**	**88**
Activity	Kayaking	Lunges	MilitaryParade	Mixing Batter	Nun chucks
Accuracy	**96**	**95**	**86**	**88**	**95**
Activity	Playing Piano	Playing Tabla	Pizza Tossing	Pole Vault	Pommel Horse
Accuracy	**95**	**86**	**88**	**95**	**86**
Activity	Pull Ups	Punch	Push Ups	Rockclimbing Indoor	RopeClimbing
Accuracy	**88**	**98**	**86**	**88**	**95**
Activity	Rowing	Salsa Spins	Skateboarding	Skiing	Skijet
Accuracy	**86**	**88**	**95**	**86**	**88**
Activity	SoccerJuggling	Swing	Playing Tabla	TaiChi	Tennis Swing
Accuracy	**95**	**95**	**86**	**88**	**95**
Activity	Trampoline Jumping	Playing Violin	Volleyball Spiking	Walking with a dog	Yo Yo
Accuracy	**95**	**86**	**88**	**96**	**86**

Significant values are in [bold].

**Figure 3:**
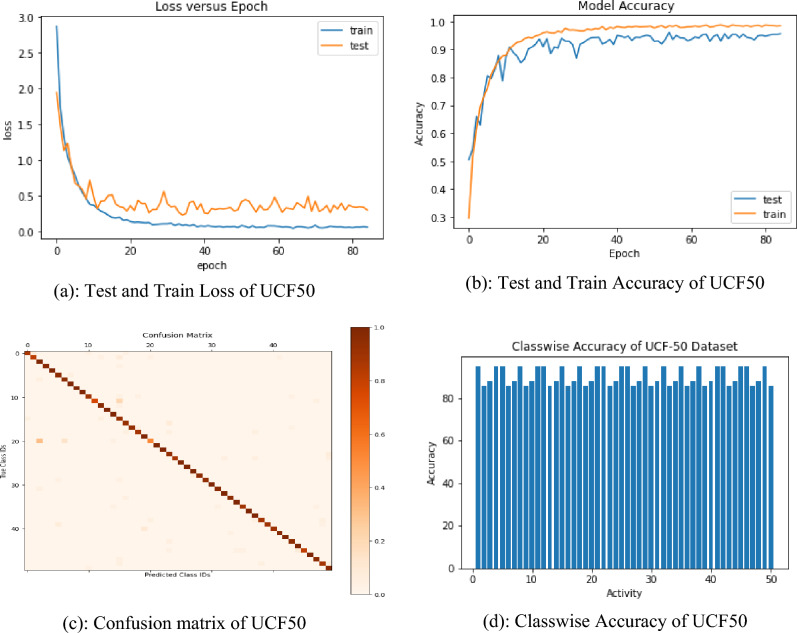
(**a**) Test and Train Loss of UCF50. (**b**) Test and Train Accuracy of UCF50. (**c**) Confusion matrix of UCF50*.* (**d**) Classwise Accuracy of UCF50.

Figure 3a shows the variation of loss of training and testing, and Fig. 3b the accuracies gained by training and testing. The confusion matrix of the trial is shown in Fig. 3c and d, which exhibit class-wise accuracies, with most of the classes reporting accuracy results of more than 94%.

### Result of UCF-Crime

UCF-Crime is a collection of long, uncut surveillance films that focus on five real-life occurrences: Abuse, Arrest, Arson, Assault, and Fighting. These anomalies were chosen because they are dangerous to the public. Each activity has 50 instances of different scenarios at different places. Each instance of video has more than 2200 frames. The suggested method is compared to five activity recognition algorithms namely I3D Siamese ^42^ C3D Siamese ^5^, DACM (without NL) ^43^, DACM (with NL) ^43^, and CNN + LSTM ^44^ using the UCF-crime dataset.

Table 6 shows that the proposed approach out performed I3D Siamese^45^, C3D Siamese^5^, DACM (without NL) ^43^, DACM (with NL) ^43^, and CNN + LSTM ^44^ for this dataset, with an accuracy of 28.8%, 31.5%, 34.1%, 35.1%, and 44.67% respectively. From the accuracy reached by CNN + LSTM, DACM, I3DSiamese, the proposed approach enhanced accuracy by 4.37%, 13.95%, 14.95%, 17.54%, and 22.24% respectively.

**Table 6:** Accuracy of the previous model compared with the proposed model.

Method	Accuracy (%)
3D CNN Siamese^45^	28.8
C3D CNN Siamese ^5^	31.5
Discriminative Anomalous Clip Miner (without NL) ^43^	34.1
Discriminative Anomalous Clip Miner (NL) ^43^	35.1
CNN + LSTM ^44^	44.67
Our Proposed	61.04

**Figure 4:**
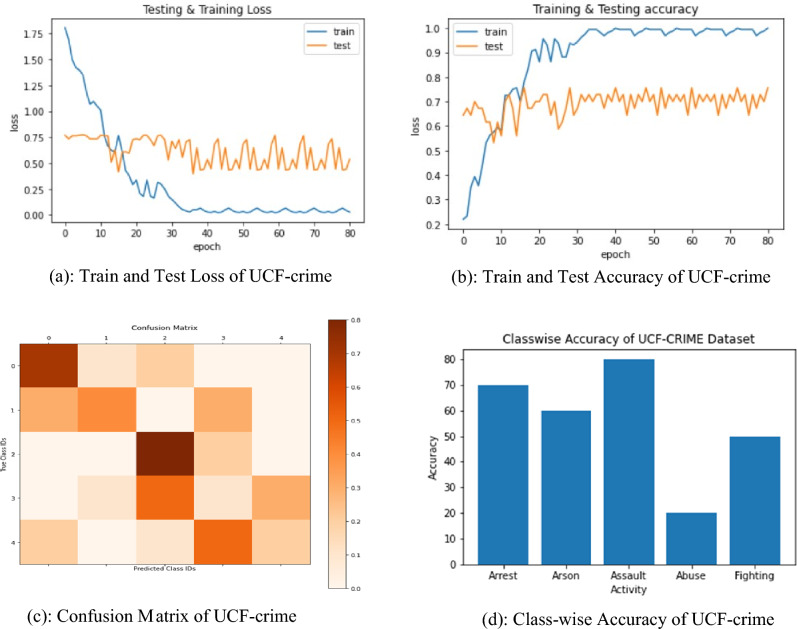
(**a**) Train and test loss of UCF-crime. (**b**) Train and test accuracy of UCF-crime. (**c**) Confusion matrix of UCF-crime. (**d**) Class-wise accuracy of UCF-crime.

Figure 4a shows the variation of loss of training and testing, Fig. 4b the accuracies gained by training and testing. The confusion matrix of the testing is exposed in Fig. 4c and d, which exhibit class-wise accuracies, where Assault activity shows 84% accuracy while Abuse shows 24% accuracy.

Table 7 shows the class-wise accuracy of activity where abuse is detected with minimum 24% accuracy while Assault is predicted with an accuracy of 84%. These activities are uncut, long real-time. Proposed method perform better than state-of-art methods.

**Table 7:** Class-wise accuracy of activity on sub-UCF Crime.

Activity	Arrest	Arson	Assault	Abuse	Fighting
Accuracy	75	65	84	24	56

Different versions of Inception and Bi-LSTM were applied to the UCF 11, UCF-Crime, and UCF-50 datasets for measure the performance of the proposed model. In our experiments, we investigate multiple modelling options: InceptionV2-LSTM, InceptionV3-LSTM, and InceptionV4-LSTM. Table 8 demonstrates the accuracy of the proposed model using the UCF 11, subUCF-Crime, and UCF-50 datasets.

**Table 8:** Performance summary of sequential model with CNN + LSTM.

Method	UCF-11	UCF-50	UCF CRIME
InceptionV2 + Bi-LSTM	95.21%	92.50%	43.60%
InceptionV4 + Bi-LSTM	95.89%	93.05%	44%
InceptionV3 + Bi-LSTM	96.16%	94%	44.50%
InceptionV3 + Bi-LSTM + attention	**98.90**	**96.04%**	**61.04%**

Significant values are in [bold].

**Figure 5:**
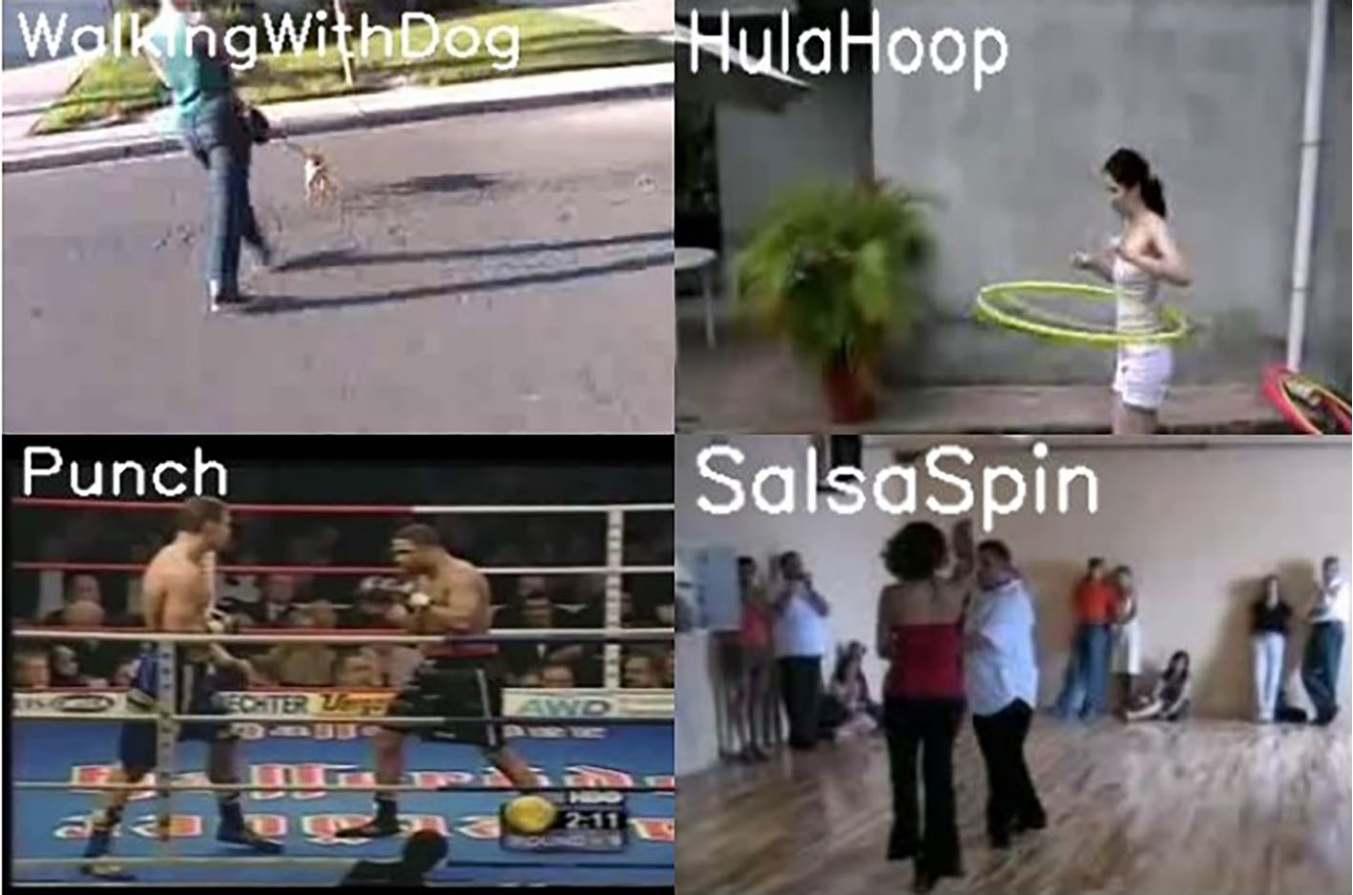
Classification on test video.

Figure 5 shown the different activity for activities detected accurately for training video.

## Conclusion

This paper proposes a deep learning architecture that uses pre-train CNN, Bi-LSTM, and attention mechanism in combination to automatically extract Spatiotemporal features from video signals and classify these video streams as normal or abnormal. The introduction of a pre-train convolutional network and Bi-LSTM enables the model to capture both long-term and local dependencies in consecutive data. The proposed architecture employs the InceptionV3 pre-train convolutional neural network to enhance feature extraction by capturing multiple local dependencies, and the Bi-LSTM is used to accurately recognize aberrant behaviors such as Abuse, Arrest, Fighting, Arson, and Assault. When compared to other competing schemes, the framework surpassed them in every significant criterion. The total rates of the accuracy of the proposed algorithm for recognizing aberrant human activity are 98.90%, 98.04%, and 61.04% with UCF11, UCF50, and subUCF crime datasets respectively. Future research could concentrate on refining the technique proposed for recognizing anomalous human behavior in distorted video feeds, which are typical of videos captured with a moving camera. Hand held devices should also consider the compatibility of their hardware with real-time data.

## Data Availability

The data utilized is publicly available at: UCF Crime Dataset|Kaggle, UCF50- Action Recognition Data Set—Center for Research in Computer Vision, CRCV|Center for Research in Computer Vision at the University of Central Florida (ucf.edu). Dataset is available on the Internet.
